# Surgery in patients with infective endocarditis and prognostic importance of patient frailty

**DOI:** 10.1007/s15010-024-02262-5

**Published:** 2024-04-27

**Authors:** Peter Laursen Graversen, Lauge Østergaard, Morten Holdgaard Smerup, Jarl Emanuel Strange, Katra Hadji-Turdeghal, Marianne Voldstedlund, Lars Køber, Emil Fosbøl

**Affiliations:** 1 Department of Cardiology, Copenhagen University Hospital – Rigshospitalet, Inge Lehmanns Vej 7, 2100 Copenhagen, Denmark; 2 Department of Cardiothoracic Surgery, Copenhagen University Hospital – Rigshospitalet, Copenhagen, Denmark; 3 Department of Data Integration and Analysis, Statens Serum Institut, Copenhagen, Denmark; 4 Department of Clinical Medicine, University of Copenhagen, Copenhagen, Denmark

**Keywords:** Infective endocarditis, Frailty, Surgery, Rehospitalization, Mortality

## Abstract

**Purpose:**

Surgery is required in 20–50% of patients with infective endocarditis (IE). Frailty increases surgical risk; however, the prognostic implications of frailty in patients undergoing IE-related surgery remain poorly understood. We aimed to assess the association between frailty and all-cause mortality or rehospitalization after discharge (≥ 14 days).

**Methods:**

We identified all IE patients who underwent surgery during admission (2010–2020) in Denmark. The Hospital Frailty Risk Score was used to categorize patients into two frailty risk groups, patients with low frailty scores (< 5 points) and frail patients (≥ 5 points). We analyzed time hospitalized after discharge and all-cause mortality from the date of surgery with a one-year follow-up. Statistical analyses utilized the Kaplan–Meier estimator, Aalen–Johansen estimator, and the Cox regression model.

**Results:**

We identified 1282 patients who underwent surgery during admission, of whom 967 (75.4%) had low frailty scores, and 315 (24.6%) were frail. Frail patients were characterized by advanced age, a lower proportion of males, and a higher burden of comorbidities. Frail patients were more hospitalized (> 14 days) in the first post-discharge year (19.1% vs.12.3%) compared to patients with low frailty scores. Additionally, frail patients had higher rates of all-cause mortality including in-hospital deaths (27% vs. 15%) and rehospitalizations (43.5% vs 26.1%) compared to patients with low frailty scores. This was also evident in the adjusted analysis (hazard ratio 1.36 [CI 95% 1.09–1.71]).

**Conclusion:**

Frailty was associated with an ≈40% increased rate of rehospitalization (≥ 14 days) or death. Further studies are needed to assess the effectiveness of surgery with a focus on frailty to improve prognostic outcomes in these patients.

**Supplementary Information:**

The online version contains supplementary material available at 10.1007/s15010-024-02262-5.

## Introduction

Frailty is characterized by a state of increased vulnerability to poor resolution of homeostasis triggered by stressors, which increases the risk of poor health outcomes [1] and has been increasingly recognized as an important risk factor associated with poor outcomes after heart valve surgery [2]. Infective endocarditis (IE) is a severe disease, where surgery is required in 20–50% of cases [3–5], and up to one-third of patients are elderly with a high burden of comorbidities [6–8]. Frailty may add to this risk, yet we have sparse data on the importance of frailty per se in this significant setting of severe disease. We need to better understand the relationship between patient frailty and surgical outcomes in IE to properly assess and select patients for surgery. This underlines the importance of choosing the right patients for surgical strategies to avoid futile surgeries. This also becomes increasingly important as surgical techniques improve and patients with IE present in more complex clinical settings (more re-do surgeries, comorbidities, and transcatheter valves) [3, 9, 10].

We hypothesized that frail patients who underwent surgery for IE were associated with an increased rate of mortality and rehospitalization. Hence, the aims of this study were to characterize patients according to frailty and assess the association of frailty with all-cause mortality and rehospitalization within the first year after surgery. Discussion of the futility of IE surgery or prevention of severe adverse outcomes after surgery is relevant, especially if high long-term mortality is evident.

## Methods

### Data sources

Danish citizens are provided with a unique personal identification number at birth, which allows for cross-linkage between different administrative registries at an individual level. The following administrative registries were used in this study: The Civil Registration System contains information on date of birth, sex, and migration status. The Danish National Patient Registry contains information on hospitalization since 1977, including diagnosis codes, date of admission, and discharge. The International Classification of Diagnosis, 10th edition (ICD-10) was added in 1994, and surgical procedures were added to the registry in 1996. The Danish Prescription Registry contains information on the dates of prescription redemption, drug type according to the Anatomical Therapeutic Chemical classification system, and drug strength. The Danish Register of the Cause of Death contains information on the date of death, including the primary and secondary causes of death registered by a physician. The Danish Microbiology Database (MiBa) contains copies of the final report from all clinical microbiology departments in Denmark, including both positive and negative cultures, since January 2010 [11]. MiBa was used to collect information on all available blood cultures. Danish administrative registries are complete, validated [12], and described in detail elsewhere [13–16].

### Study population

**Fig. 1:**
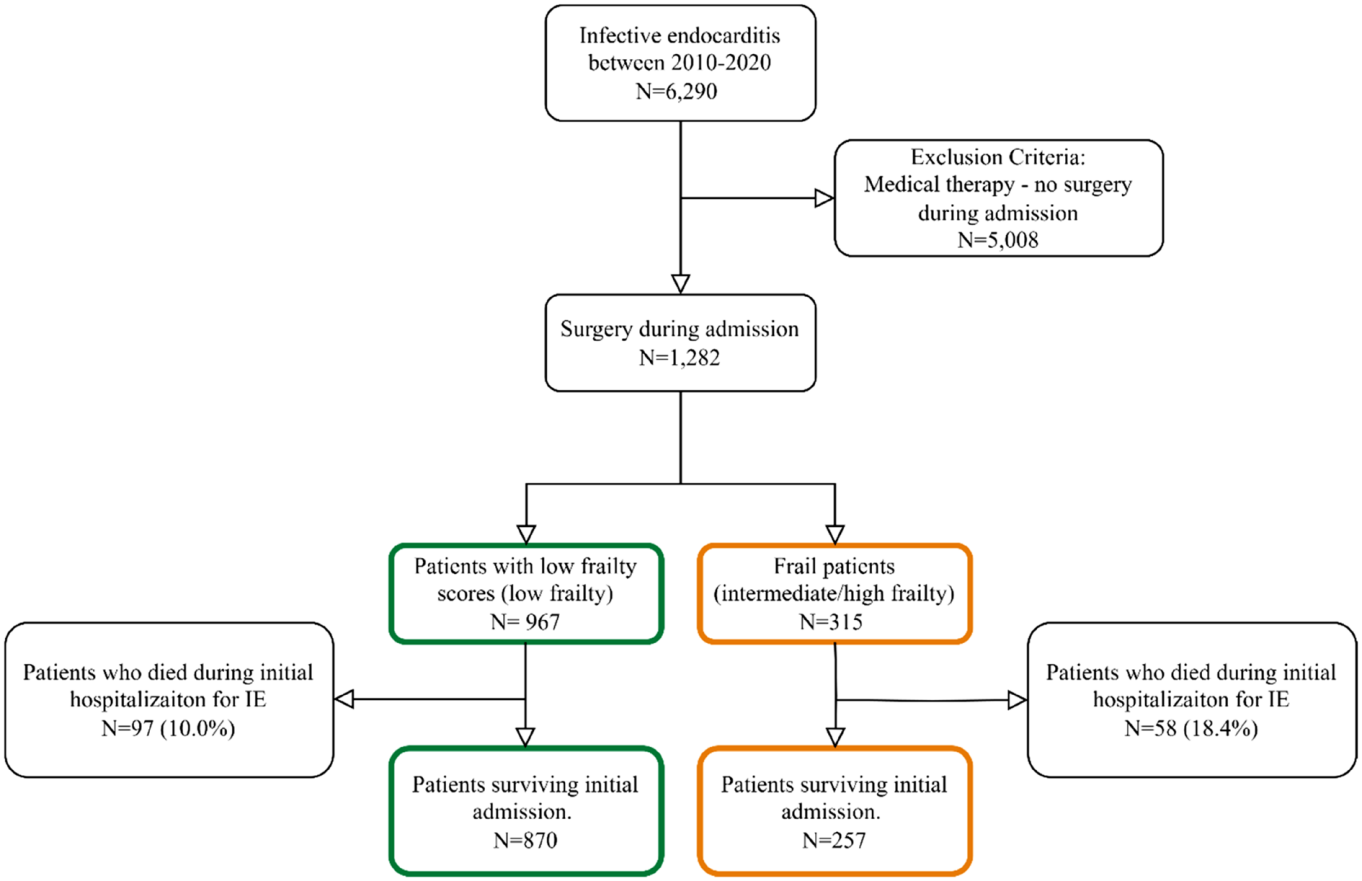
Flowchart of the study population

We identified all patients with the first-time episode of IE between 2010 and 2020 who underwent surgery during admission using the following ICD-10 diagnosis codes for IE: DI33, DI38, and DI39.8. We defined IE using the following criteria to increase the accuracy of the ICD-10 diagnosis codes: patients hospitalized for ≥ 14 days or patients who died within the first two weeks of admission. These criteria for IE have been validated with a positive predictive value of 90% [12, 17]. To identify patients who underwent surgery during initial admission, we used procedural codes for surgical intervention on one or more heart valves during admission (see Supplementary Table S1 for procedure codes). IE patients who did not undergo surgery were excluded (Fig. 1).

### Definition of frailty

To categorize patients into frailty risk groups, we used the Hospital Frailty Risk Score (HFRS), a frailty risk assessment tool based on 109 ICD-10 diagnosis codes internally and externally validated among elderly patients in the United Kingdom (Supplementary Table S2 shows ICD-10 codes) [18]. Each ICD-10 code is acquainted with a number of points and the HFRS is a summarization of all the points that range from 0 to > 100. Information from prior hospital admissions up to 10 years before the date of IE surgery, a score was calculated for each patient as done previously [19]. Patients were stratified into two groups according to frailty: patients with low frailty scores (frailty scores < 5) and frail patients (intermediate/high frailty, frailty scores ≥ 5). We divided frailty into two risk groups due to a limited study population, especially for highly frail individuals. This study aimed to comprehensively evaluate frailty with a broader definition to minimize the risk of misclassification.

### Comorbidities, medication, and microbiological etiology

Comorbidities were defined using ICD-10 primary and secondary diagnosis codes within ten years of the index date from the Danish National Patient Registry. We included all diagnoses from hospitalizations and outpatient visits. Medication was defined as a prescription redeemed for a specific drug group within six months of the index date. Hypertension was defined as the redemption of prescriptions for at least two different types of antihypertensive medication. Diabetes was defined as either redemption of a prescription for glucose-lowering medication or a diagnosis code for diabetes (Supplementary Table S1). The microbiological etiology was identified as a positive blood culture collected within 30 days of the index date and until the discharge date of IE to account for diagnostic delay derived from MiBa. To identify the most likely microbiological cause of IE, microbiological etiologies were ranked and categorized in groups as described previously [20].

The specific microorganisms by the parent groups of microbiological etiologies are shown in Supplementary Table S3a-c.

### Outcomes and follow-up

To assess the association of frailty with severe prognostic outcomes, we defined a composite primary outcome as the first of either all-mortality or rehospitalization for ≥ 14 days within the first year from the date of surgery. The secondary outcomes comprised the individual components of the primary composite outcome and in-hospital mortality. Patients were followed from the date of surgery until either one year after, date of death, date of two weeks of hospitalization after the initial IE admission, date of emigration, or censoring (December 31, 2021), whichever came first. In the analysis of rehospitalization alone, patients who died during initial hospitalization for IE were excluded (*N* = 155) and follow-up began at the date of discharge.

### Statistical analysis

Baseline characteristics were compared and stratified by frailty groups. Categorical variables were reported in numbers and percentages and continuous variables in median with corresponding 25th and 75th percentiles. Comparisons between the two groups were performed using Pearsons’s chi-square test for categorical variables and the Wilcoxon rank sum test for continuous variables. To depict the burden of rehospitalization in the first year after IE discharge by frailty, patients were categorized according to days hospitalized: “Never admitted”, “1–14 days”, “15–28 days”, and “ > 28 days”. A final category “Died” comprised patients who died during the first year after IE discharge regardless of any hospitalizations. Furthermore, we conducted a sensitivity analysis, where patients only were categorized according to days hospitalized not including death during the first year.

To assess the association of frailty with in-hospital mortality, a multivariable logistic regression analysis was conducted, adjusting for the following covariates not included in the HFRS: age, sex, microbiological etiology, hypertension, ischemic heart disease, heart failure, diabetes, liver disease, and malignancy. Age was included as a continuous variable and assessment of linearity was found to be valid on the log scale.

Crude rates of mortality or rehospitalization for the composite outcome (comprising all-cause mortality or rehospitalization ≥ 14 days) and all-cause mortality based on frailty were calculated using the 1-Kaplan–Meier estimator. For rehospitalization, the crude rehospitalization rates were calculated using the Aalen–Johansen estimator with all-cause mortality as a competing risk. The association between frailty and the primary composite outcome of one-year mortality or rehospitalization ≥ 14 days, as well as the secondary outcomes (all-cause mortality or rehospitalization), was examined using the Cox proportional hazards regression model. The cause-specific Cox model was utilized when assessing the adjusted association between frailty and rehospitalization treating all-cause mortality as censoring. Adjustments for covariates in this model mirrored those used in the logistic regression model. To verify the proportional hazard (PH) assumption, we conducted tests using accumulated residuals (Martingales and Schoenfeld residuals), and the assumption was found to be valid for all variables included in the final models. Age was incorporated into the models as a continuous variable, and its functional form, assessed using Martingale residuals, was determined to be linear on the log scale.

A *P*-value < 0.05 was considered statistically significant. Data management and survival analyses were conducted in SAS Enterprise 7.1 (SAS Institute, Inc., Cary, NC, USA), and figures and tables were performed using R software version 4.2.1 (R Foundation for Statistical Computing, Vienna, Austria).

### Sensitivity analyses

To further elucidate frailty, patients were categorized into the following three frailty risk groups: low frailty risk (< 5), intermediate frailty risk (5–15 points), and high frailty risk (> 15 points) as originally done [18]. The HFRS has been validated in elderly patients (≥ 75 years of age). Thus, we conducted a subgroup analysis of elderly patients (≥ 75 years of age) with frailty in two categories to investigate the association between frailty and mortality and rehospitalization in elderly patients. All sensitivity analyses were performed using the same statistical methods as described previously. The PH assumption of the Cox regression model was tested with accumulated residuals and found to be valid for all variables included in the final models.

## Results

### Baseline characteristics of the study population

We identified 1282 patients with first-time IE who underwent surgery during admission. Of those, 1189 (92.8%) underwent left-sided valve surgery, 31 (2.4%) underwent right-sided surgery, and 62 (4.8%) underwent combined left-sided and right-sided surgery (Supplementary Table S4). Patients were categorized according to frailty risk groups: 967 (75.4%) low frailty (median frailty score 1.1 [0–2.5]), 270 (21.1%) intermediate frailty (median frailty score 7.4 [6.2–9.7]), and 45 (3.5%) high frailty (median frailty score 17.6 [16.2–21.9]). The number of frail patients was 315 (24.6%) defined as either intermediate or high frailty using the HFRS (median frailty score 7.9 [6.4–11.2]). Frail patients were older (67.0 [53.0–72.0] vs. 64.0 [56.5–73.0] years) and had a lower proportion of males (71.7% vs 77.9%). Furthermore, frail patients had a higher burden of comorbidities, including dialysis and medication use prior to admission, than patients with low frailty scores. *Staphylococcus aureus* (*S. aureus*) and *Enterococcus* species (spp.) identified as the primary causative microorganisms were more prevalent among frail patients compared to patients with low frailty scores (27.1% vs. 19.4% and 22.0% vs. 13.0%, respectively; see Table 1). In patients who survived the initial hospitalization for IE (*N* = 1127) similar findings were observed (Supplementary Table S5).

**Table 1:** Baseline characteristics according to frailty

Covariates	Patients with low frailty scores, *N* = 967	Frail patients, *N* = 315	*p*-value
Cumulative frailty score	1.1 [0–2.5]	7.9 [6.4–11.2]	< 0.001
Males	753 (77.9%)	226 (71.7%)	0.026
Age (years)	64.0 [53.0–72.0]	67.0 [56.5–73.0]	0.004
Length of hospital stay (days)	45.0 [34.0–57.0]	48.0 [40.0–64.0]	< 0.001
Microbiological etiology*			< 0.001
*S. aureus*	187 (19.4%)	85 (27.1%)	
*Streptococcus* spp.	381 (39.6%)	89 (28.3%)	
*Enterococcus* spp.	125 (13.0%)	69 (22.0%)	
CoNS	72 (7.5%)	26 (8.3%)	
Other	57 (5.9%)	14 (4.5%)	
Negative	140 (14.6%)	31 (9.9%)	
Prior prosthesis	158 (16.3%)	91 (28.9%)	< 0.001
Cardiac implantable electrical devices	46 (4.8%)	25 (7.9%)	0.032
Aortic valve disease	244 (25.2%)	122 (38.7%)	< 0.001
Mitral valve disease	83 (8.6%)	43 (13.7%)	0.009
Atrial fibrillation	120 (12.4%)	71 (22.5%)	< 0.001
Heart failure	97 (10.0%)	63 (20.0%)	< 0.001
Ischemic heart disease	134 (13.9%)	93 (29.5%)	< 0.001
Hypertension	343 (35.5%)	172 (54.6%)	< 0.001
Chronic kidney disease	24 (2.5%)	54 (17.1%)	< 0.001
Dialysis	18 (1.9%)	35 (11.1%)	< 0.001
Diabetes	116 (12.0%)	86 (27.3%)	< 0.001
Liver disease	14 (1.4%)	21 (6.7%)	< 0.001
Chronic obstructive pulmonary disease	44 (4.6%)	33 (10.5%)	< 0.001
Malignancy	72 (7.4%)	40 (12.7%)	0.004
Charlson comorbidity index			< 0.001
0	634 (65.6%)	76 (24.1%)	
1–2	276 (28.5%)	140 (44.4%)	
2	57 (5.9%)	99 (31.4%)	
Anticoagulants	157 (16.2%)	104 (33.0%)	< 0.001
Beta blockers	217 (22.4%)	122 (38.7%)	< 0.001
Lipid-lowering medication	262 (27.1%)	151 (47.9%)	< 0.001
RAS inhibitors	313 (32.4%)	154 (48.9%)	< 0.001

*6 Missing values

Categorical variables were reported with frequency and percentages and continuous variables with median and 25th–75th percentiles. To assess the statistical difference between the two groups, we used Pearson’s Chi-square test for categorical variables and the non-parametric Wilcoxon rank sum test for continuous variables

### Time hospitalized the first year after discharge according to frailty

**Fig. 2:**
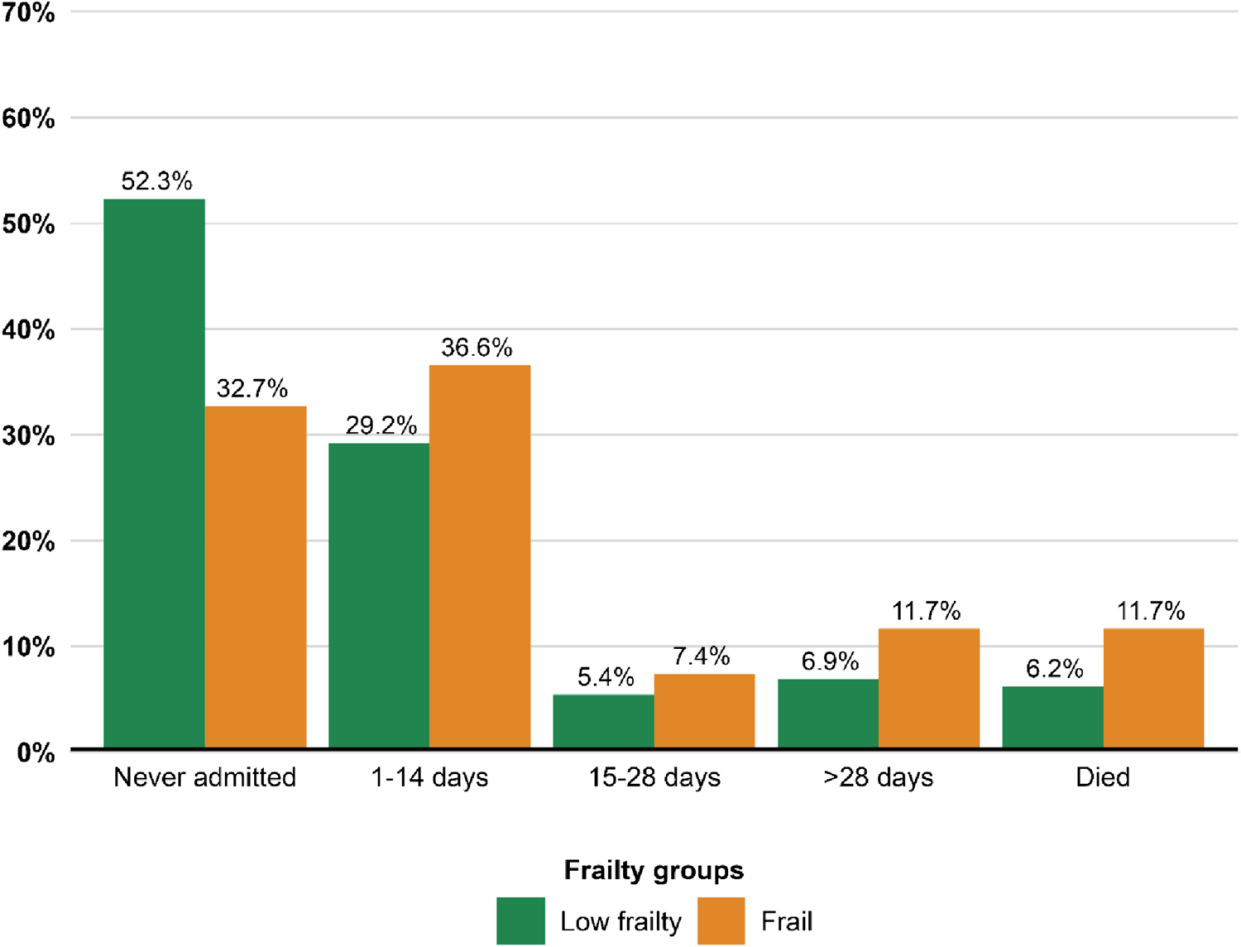
Days hospitalized or mortality one year from IE discharge according to frailty. Patients who survived initial hospitalization for IE (*N* = 1127) stratified by frailty and categorized according to days hospitalized after IE discharge or all-cause mortality. The y-axis shows the percentage and percentages above the bars sum up to 100% for each frailty group, e.g., all green bars sum up to 100%

Frail patients surviving the initial admission spent more time hospitalized compared with patients with low frailty scores: In frail patients, 19.1% were hospitalized for > 14 days compared with 12.3% in patients with low frailty scores. Approximately, one-third (32.7%) of frail patients survived for one year without any rehospitalizations compared to more than half (52.3%) of the patients with low frailty scores (Fig. 2). Evaluating only the time spent in the hospital in patients surviving initial admission irrespective of death during the first year after IE discharge, 25.7% of frail patients were admitted for > 14 days compared to 15.3% of patients with low frailty scores (Supplementary Fig. S1).

### In-hospital mortality according to frailty

During the initial hospitalization for IE, 97 patients with low frailty scores died (10.0%) compared with 58 frail patients (18%). In the adjusted analysis, frail patients were associated with an odds ratio of 1.45 (95% CI 0.99–2.13, *p* = 0.057) for in-hospital mortality compared to patients with low frailty scores.

### One-year risk of mortality or rehospitalization stratified by frailty

**Fig. 3:**
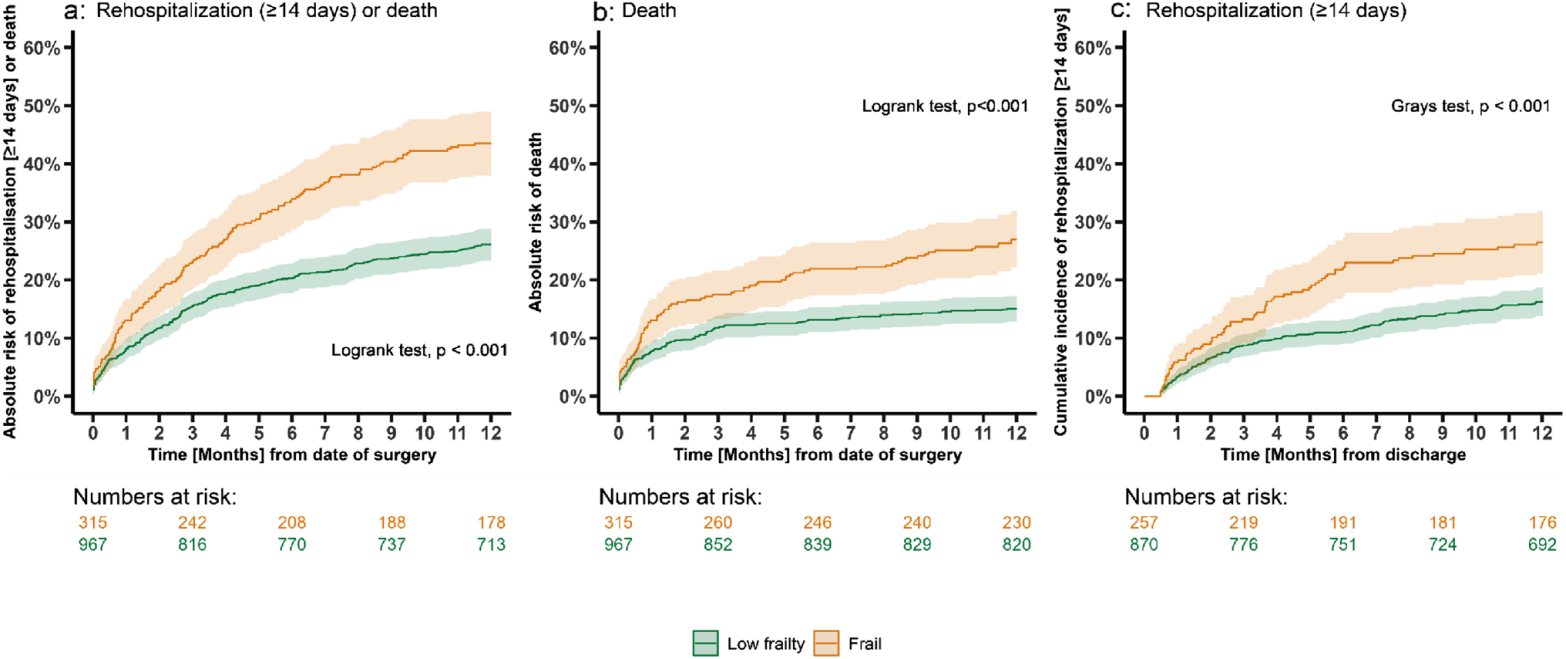
**a**–**c** Risk of rehospitalization or death according to frailty during 12 months of follow-up. Legend: **a**1-KM estimates depicting the crude absolute risk at one year of rehospitalization for ≥ 14 days or death and **b** 1-KM estimates showing crude mortality rates at one year. **c** Aalen–Johansen estimates showing the cumulative incidence of rehospitalization (≥ 14 days) for patients who survived initial admission (*N* = 1127) at one year with all-cause mortality as a competing risk. Patients with low frailty scores are depicted with a green color and frail patients are depicted with an orange color. The shaded areas represent 95% confidence intervals. The x-axis represents the number of months since the date of surgery or the date of discharge and the y-axis represents the absolute risk in percentages. Overall comparison of the frailty groups was performed using the Logrank test for a and b and the Gray’s test for c

**Fig. 4:**
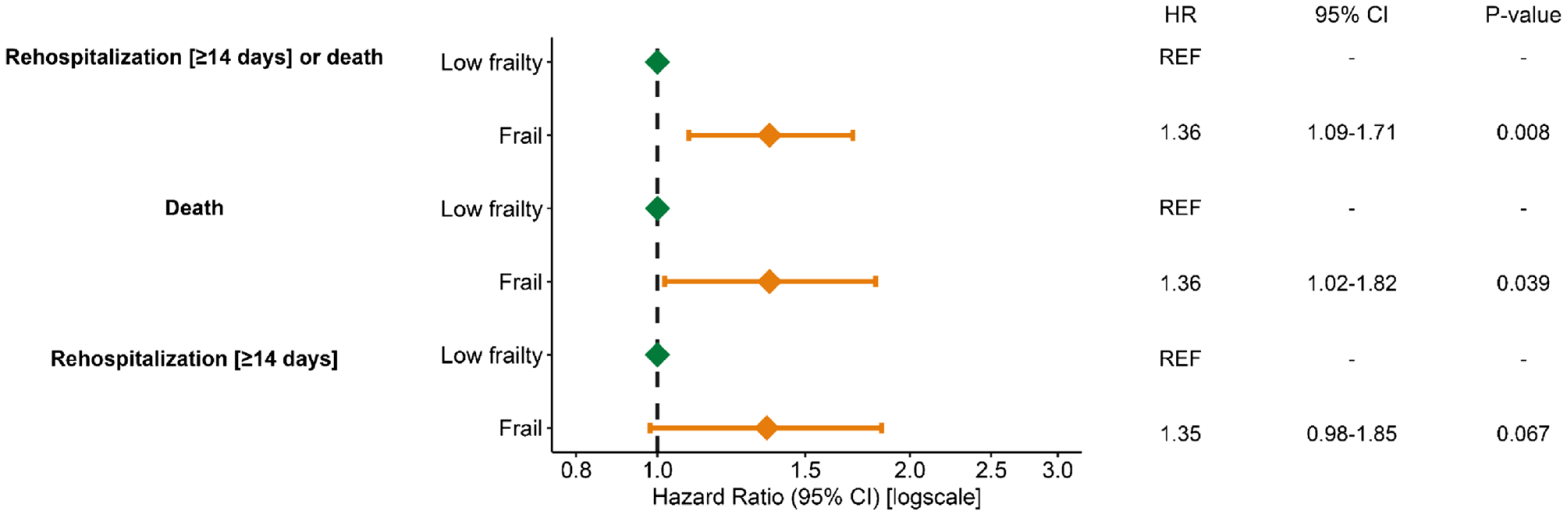
Associated rate of the composite outcome of rehospitalization or mortality and mortality alone for frail patients compared to patients with low frailty scores. Legend: Multivariate Cox proportional hazards model with hazard ratios and 95% confidence levels adjusted for the following covariates: age, sex, microbiological etiology, hypertension, ischemic heart disease, heart failure, diabetes, liver disease, and malignancy. We used the cause-specific Cox model to test the association between frailty and rehospitalization (≥ 14 days) for patients surviving the initial admission for IE (*N* = 1127). Patients were followed from either the date of surgery or the date of discharge. *HR* hazard ratio. 95% *CI* 95% confidence levels

During 12 months of follow-up, the primary outcome of all-cause mortality or rehospitalization for ≥ 14 days occurred in 252 out of 967 patients with low frailty scores (321.2 events per 1000 person-years [PY]) compared to 137 out of 315 frail patients (629.8 events per 1000 PY). To see a full summary of events, cumulative follow-up in person-years, and crude event rates for all outcomes see Supplementary Table S6. Frail patients had a higher absolute risk of 43.5% (95% CI 37.7–48.7%) for death or rehospitalization (≥ 14 days) compared to 26.1% (95% CI 23.3–28.8%, *p* < 0.001) in patients with low frailty scores. Frail patients also had an increased mortality rate of 27.0% (95% CI 21.9–31.7%) compared to 15.0% (95% CI 12.7–17.2%, *p* < 0.001) in patients with low frailty scores (Fig. 3a–c). In adjusted analysis, frail patients were associated with an increased rate of the primary composite outcome of death or rehospitalization (HR 1.36 [CI 95% 1.09–1.71], *p* = 0.008) compared to patients with low frailty scores. Evaluating the secondary outcomes, we observed that frail patients were associated with increased rates of mortality (adjusted HR 1.36 [CI 95% 1.02–1.82], *p* = 0.039) and rehospitalizations (adjusted HR 1.35 [CI 95% 0.98–1.85, *p* = 0.067) compared to patients with low frailty scores (Fig. 4).

### Sensitivity analyses

Evaluating frailty in three frailty risk groups (low, intermediate, and high), the comorbidity burden, *S. aureus* and *Enterococcus* spp. IE increased incrementally with frailty level (Supplementary Table S7). An incremental increase in days hospitalized or mortality within the first year after discharge was found with increasing levels of frailty in patients surviving the initial admission (Supplementary Fig. 2). Similar findings were observed evaluating the rates of rehospitalization or all-cause mortality at one year after either date of surgery or discharge (Supplementary Fig. S3a–c). In the adjusted Cox regression model, high frailty was associated with a more than two-fold higher rate of rehospitalization or death (HR 2.52 [95% CI 1.69–3.75], *p* < 0.001) and intermediate frailty was associated with a 21% increased rate of rehospitalization or death, although not statistically significant (HR 1.21 [95% CI 0.95–1.55], *p* = 0.118), compared to patients with low frailty scores. In elderly patients aged  ≥75 years (*N* = 217), frail patients (*N* =62 [28.6%]) had a higher burden of comorbidities and use of lipid-lowering medication prior to admission compared to patients with low frailty scores (*N* = 155 [71.4%]). Microbiological etiologies were similar to those of the entire cohort, although the prevalence of *Enterococcus* spp. was higher (Supplementary Table S8). In addition, we assessed the rate of rehospitalization or death within the first year after surgery and found no association between frailty and the rate of rehospitalization or death in this subgroup.

## Discussion

The aims of this retrospective observational nationwide study were to characterize patients with IE, who underwent surgery during initial admission according to frailty and to assess the association between frailty and mortality or rehospitalization. This study had two main findings. First, we observed that one out of four patients were characterized as frail using the HFRS with a higher burden of comorbidities, *S. aureus* and *Enterococcus* spp. Second, frail patients were associated with approximately 40% higher rate of death and rehospitalization in the first year after surgery compared to patients with low frailty scores. In sensitivity analyses, an incremental increase in the rate of rehospitalization and mortality according to frailty risk groups was observed with a more than two-fold increase in the rate of mortality or rehospitalization for high frailty compared to low frailty.

The importance of frailty has been increasingly recognized in patients undergoing planned cardiac surgery, over the last decade. Previous studies have found that the prevalence of frail patients (intermediate or high frailty) utilizing the HFRS ranged between 39% and 49% for different cardiac surgical procedures (left atrial appendage closure, ablation for atrial fibrillation, mitral valve repair or transcatheter aortic valve implantation [TAVI]) [21–24]. Other studies applying other frailty assessment tools including a meta-analysis observed the prevalence of frail patients undergoing major cardiac surgery ranged between 4% and 85% [2, 25]. The prevalence of frailty significantly differs among countries, with a more than two-fold higher occurrence of high frailty observed among TAVI patients in the US compared to Denmark [23, 24], as assessed by the HFRS. Frailty assessment in IE cohorts has been sparsely reported. Previous studies have reported that 45% of patients with native valve IE were classified as frail according to the HFRS (intermediate or high frailty), with higher prevalence in females and patients with IE after prosthetic valve, especially among TAVI patients [26, 27]. The evaluation of frailty presents challenges and is greatly influenced by the choice of the frailty assessment tool and the characteristics of the studied population. This study enlightens that the prevalence of frailty was different compared to other studied populations, most likely because of the highly selected patients who were younger with a lower burden of comorbidities. Further, IE patients undergoing surgery are not likely comparable because of the disease course and the acute/urgent setting in which surgery was performed in these patients. Although this study comprised highly selected patients from the heart team based on the pre-operative risk assessment, a considerable proportion of patients who underwent surgery for IE were still classified as frail. Previous studies from population-based cohorts have characterized patients with IE with a considerable burden of comorbidities [20, 28, 29], and the highest incidence among patients > 60 years [4, 28, 30]. Hence, the proportion of frail patients in this study is not surprising.

### Mortality and rehospitalization

Frail patients who underwent surgery during the initial admission for IE had a higher rate of rehospitalizations or deaths within the first year after surgery compared to patients with low frailty scores. Furthermore, frail patients were associated with higher odds of in-hospital mortality compared to patients with low frailty scores, although this was only borderline significant. This may be the result of a relatively small study population with few events. In elderly patients, we did not find any differences in the cumulative incidences of rehospitalization or death according to frailty. This may, in part be explained by a small sample size and a highly selected patient population of only the healthiest and non-frail patients or patients with a strong recommendation for surgery. Similar findings have been observed in other cardiac procedures such as transcatheter valve replacement or repair [23], percutaneous left atrial appendage closure [22], and ablation for atrial fibrillation [21]. This underlines the importance of improving the risk stratification of patients to reduce the burden of rehospitalization and mortality in patients undergoing cardiac surgery.

### The potential role of frailty in pre-operative risk assessment for IE patients

Current IE guidelines lean towards surgery of patients with left-sided endocarditis and recommend urgent surgery (Class I) within three to five days in case of uncontrolled infection, heart failure with poor hemodynamic tolerance, and high risk of embolization (vegetation size ≥ 10 mm combined with either one or more embolic events despite appropriate antibiotic therapy or other indication for surgery) [31]. However, IE patients become more complex regarding comorbidity burden, re-do surgeries, and the increasing use of transcatheter valves. This underlines the significance of carefully weighing the efficacy of surgery against potential risks. Over the last decade, multitude of risk scores for IE has been developed; however, the lack of accuracy and validity limits their clinical utility [32–36]. Furthermore, the performance of the risk scores has varied substantially depending on the different cohorts in which they were tested [32, 36–38]. Thus, implementation of systematic evaluation of frailty in the pre-operative risk assessment with other risk assessment tools such as EuroSCORE II, RISK-E, AEPEI, or STS-IE may improve the risk stratification of patients to better identify patients in high risk of severe adverse outcomes. Frailty assessment tools including HFRS only acceptably predict outcomes [2]; however, the addition of frailty in surgical risk scores has been shown to improve risk stratification and identify patients at high risk for coronary artery bypass grafting or/and valve surgery [39]. Optimizing the condition prior to surgery or postponing non-emergency surgery to improve the presurgical condition, could potentially improve outcomes after surgery. In patients with need of emergency surgery or a class I indication for surgery, assessment of frailty to improve pre-surgical condition would be less useful because of the disease course and the unacceptable high risk of mortality if surgical treatment is not performed or postponed [5, 40]. However, frailty is also an important factor in the assessment of surgical futility; thus, it should be incorporated into the shared decision making and conversation with the patient to avoid futile surgeries in patients with unacceptable high surgical risk [31].

### Strengths and limitations

This study benefits from contemporary data with minimal loss to follow-up in the Danish national registries. Additionally, it was possible to classify all patients into a frailty category using the HFRS. The registries are of high quality, and most of the diagnostic and procedural codes have been validated previously. However, this study had some limitations. Firstly, we did not have access to echocardiographic data available, including the size and location of the vegetations, signs of abscess or fistula, degree of heart valve regurgitation, left ventricular ejection fraction, right ventricular systolic function, and assessment of pulmonary hypertension; thus, important echocardiographic parameters were lacking to determine the preoperative risk of the patients. Furthermore, we did not have any information on the indications for surgery and surgical risk scores such as the EuroSCORE II or STS-IE. We did not have any information on antibiotic treatment during hospitalization. The HFRS has been validated in other European countries, but not in a Danish cohort, and the HFRS has been validated only in elderly patients (≥ 75 years of age). The HFRS is based on the ICD-10 codes; however, the severity of the disease (ICD-10 code) is not differentiated in the risk score. While the HFRS has been compared with the Rockwood Frailty Scale and Fried Frailty Phenotype, showing a fair overlap, information on other assessment tools (e.g., Clinical Frailty Scale, Fried Frailty Phenotype), muscle strength/grip strength, time up and go test (TUGT), gait speed test, use of mobility aid devices, activities of daily living (ADL), or whether the patients were self-reliant or needed assistance in daily living was not available. Lastly, the HFRS has been proven to discriminate weakly between individuals with different outcomes (30-day mortality, long length of hospital stay, and emergency readmissions) although improved by adding additional risk factors [18].

In conclusion, our contemporary nationwide data demonstrate that 25% of patients with infective endocarditis, who underwent surgery, were classified as frail. Moreover, frail patients were associated with increased rates of rehospitalization and all-cause mortality. Thus, frailty may have a role in enhancing pre-operative risk assessment to improve prognostic outcomes especially for patients without a class I indication for surgery, potentially averting futile surgeries. Future studies are warranted to clarify the effectiveness of surgery, with a focus on frailty, to improve risk stratification and prognostic outcomes in these patients.

## Supplementary Information

Below is the link to the electronic supplementary material.

Supplementary file1 (DOCX 530 KB)

## Abbreviations

CoNS: Coagulase-negative staphylococci
IE: Infective endocarditis
HFRS: Hospital Frailty Risk Score
ICD-10: International Classification of Disease, 10th edition
MiBa: The Danish Microbiology Database
*S. aureus*: *Staphylococcus aureus*
Spp.: Species
TAVI: Transcatheter aortic valve implantation
