# Supplementary material for: Surgery in patients with infective endocarditis and prognostic importance of patient frailty
Source: Infection. 2024 Apr 27;52(5):1953–63. doi: 10.1007/s15010-024-02262-5 (PMC11499324; doi:10.1007/s15010-024-02262-5)

**Supplementary Information**

| **Table S1:** Overview of codes used to define study population, medication, and comorbidity | |
| --- | --- |
| **Category** | **Codes (*Within ten years of index date)*** |
| Study population |  |
| Infective endocarditis (first-time event) | ICD-10: DI33, DI38, DI398 |
| Surgery during admission | NOMESCO |
| *Aortic valve* | Bioprostheses: KFMD10, KFCA70  Mechanical prostheses: KFMD00, KFCA60  Other valve prostheses: KFMD20, KFMD30, KFMD33, KFMD96 |
| *Mitral valve* | KFK |
| *Pulmonic valve* | KFJB, KFJC, KFJE, KFJF, KFJW |
| *Tricuspid valve* | KFG |
| Comorbidity/previous intervention (prior to index date) |  |
| Aortic valve disease | ICD-10: I35s  ICD-8: 395, 396 |
| Atrial flutter/fibrillation | ICD-10: DI48,  ICD-8:42793, 42794 |
| Chronic obstructive lung disease (COPD) | ICD-10: DJ42-44  ICD-8: 490-492 |
| Chronic kidney disease (CKD) | ICD-10: DN02-08, DN11-12, DN14, DN18-19, DN26, DN158-160, DN162-164, DN168, DQ612-613, DQ615, DQ619, DE102, DE112, DE132, DE142, DI120, DI131, DM300, DM313, DM319, DM321, DZ992  ICD-8: 403-404, 581-584, 25002, 40039, 59009, 59320, 75310, 754311, 75319 |
| CIED** | NOMESCO: BFCA0, BFCB0 |
| Diabetes mellitus | ICD-10: DE10-14  ICD-8: 250  ATC code: A10 |
| Heart failure | ICD10: DI42, DI50, DI099, DI110, DI130, DI132, DJ819  ICD-8: 425, 428, 4270, 4271, 78249 |
| Ischemic heart disease (IHD)  *PCI***  *CABG*** | ICD-10: DI20-25  ICD-8: 410-414  NOMESCO: KFNG0, KFNG10, KFNG12, KFNG96  NOMESCO: KFNA, KFNB, KFNC, KFNC, KFNE |
| Liver disease | ICD-10: DB15-19, DK70-77, DC22, DI982, DZ944, DD684C, DQ618A,  ICD-8: 571-573, 155, 070 |
| Malignancy | ICD-10: DC00-97, not including C44 (skin cancer)  ICD-8: 140-209, not including 173 (skin cancer) |
| Mitral valve disease | ICD-10: I34  ICD-8: 394, 396 |
| Prosthetic heart valve | KFKD, KFMD, KFGE, KFJF, KFCA60, KFCA70 |
| Renal dialysis | ICD-10: DZ992  NOMESCO: BFJD2 |
| Medication (within six months prior to index date) |  |
| Anticoagulants (DOAC and VKA) | ATC code: B01AA, B01AE, B01AF |
| Beta blockers | ATC code: C07, C09BX |
| Lipid-lowering medication | ATC code: C10 |
| RAS-inhibitors | ATC code: C09 |
| Hypertension  *Adrenergic antagonists*  *Beta blockers*  *Calcium channel blockers*  *Combined diuretics*  *Loop-diuretics*  *MRA*  *RAS-inhibitors*  *Thiazide diuretics*  *Vasodilating drugs* | *Defined as two or more antihypertensive drugs within six months prior to index data including the following:*  ATC code: C02A, C02B, C02C  ATC code: C07, C09BX  ATC code: C08, C07F, C09BB, C09DB  ATC code: C07C, C08G, C03B, C09BA, C09DA  ATC code: C03C, C03EB01, C03EB02  ATC code: C03DA01-C03DA04  ATC code: C09  ATC code: C03A, C07B, C07D, C09XA52, C03EA01  ATC code: C02DB, C02DD, C02DG |
| Charlson Comorbidity Index | *Within the last five years from index date* |
| Myocardial infarction | ICD-10: I21.x*, I22.x, I125.2 |
| Congestive Heart Failure | ICD-10: I109.9, I11.0, I113.0, II3.2, I125.5, I142.0, I142.5-I142.9, I143.x, I50.x, P29.0 |
| Peripheral vascular disease | ICD-10: I70.x, I71.x, I73.1, I73.8, I73.9, I77.1, I79.0, I79.2, K55.1, K55.8, K55.9, Z95.8, Z95.9 |
| Cerebrovascular disease | ICD-10: G45.x, G46.x, H34.0, I60.x-I69.x |
| Dementia | ICD-10: F00.x-F03.x, F05.1, G30.x, G31.1 |
| Chronic pulmonary disease | ICD-10: I27.8, I27.9, J40.x-J47.x, J60.x-J67.x, J68.4, J70.1, J70.3 |
| Rheumatic disease | ICD-10: M05.x, M06.x, M31.5, M32.x-M34.x, M35.1, M35.3, M36.0 |
| Peptic ulcer disease | ICD-10: K25.x-K28.x |
| Mild liver disease | ICD-10: B18.x, K70.0-K70.3, K70.9, K71.3-K71.5, K71.7, K73.x, K74.x, K76.0, K76.2-K76.4, K76.8, K76.9, Z94.4 |
|  |  |
| Moderate to severe liver disease | ICD-10: I85.0, I85.9, I86.4, I98.2, K70.4, K71.1, K71.2, K72.9, K76.5-K76.7 |
|  |  |
| Diabetes without complications | ICD-10: E10.0, E10.1, E10.6, E10.8, E10,9, E11.0, E11.1, E11.6, E11.8, E11.9, E12.0, 12.1, E12.6, E12.8, E12.9, E13.0, E13.1, E13.6, E13.8, E13.9, E14.0, E14.1, E14.6, E14.8, E14.9 |
| Diabetes with chronic complications | ICD-10: E10.2-E10.5, E10.7, E11.2-E11.5, E11.7, E12.2-E12.5, E12.7, E13.2-E13.5, E13.7, E14.2-E14.5, E14.7 |
| Renal disease | ICD-10: I12.0, I13.1, N03.2-N03.7, N05.2, N05.7, N18.x, N19.x, N25.0, Z49.0, Z49.2, Z94.0, Z99.2 |
| Hemiplegia or paraplegia | ICD-10: G04.1, G11., G80.1, G80.2, G81.x, G82.x, G83.0-G83.4, G83.9 |
| Any malignancy including lymphoma and leukemia except malignant neoplasm of the skin | ICD-10: C00.x-C26.x, C30.x-C34.x, C37.x-C41.x, C43.x, C45.x-C58.x, C60.x-C76.x, C81.x-C85.x, C88.x, C90.x-C97.x |
| Metastatic solid tumor | ICD-10: C77.x-C80.x |
| HIV/AIDS | ICD-10: B20.x- B22.x, B24.x |
| * “.x” indicates every sub-classification of diagnosis code or ATC code.  ** Any time before index date.  ICD: international classification of diseases, ATC: Anatomical Therapeutical Classification System, AMI: acute myocardial infarction, PCI: percutaneous coronary intervention, CABG: coronary artery bypass grafting, CIED: cardiac implantable electronic device, DOAC: direct oral anticoagulant therapy, MRA: aldosterone receptor antagonists, RAS: renin angiotensin system, VKA: vitamin K antagonists | |

| Table S2: ICD-10 codes used in The Hospital Frailty Risk Score  *We used information from prior Hospital admissions up to 10 years before date of IE surgery.* | | |
| --- | --- | --- |
| Diagnosis description | Diagnosis code | Points awarded |
| Dementia in Alzheimer disease | F00 | 7.1 |
| Hemiplegia | G81 | 4.4 |
| Alzheimer's disease | G30 | 4.0 |
| Sequelae of cerebrovascular disease | I69 | 3.7 |
| Other symptoms and signs involving the nervous and musculoskeletal systems (R29.6 Tendency to fall) | R29 | 3.6 |
| Other disorders of urinary system (includes urinary tract infection and urinary incontinence) | N39 | 3.2 |
| Delirium, not induced by alcohol and other psychoactive substances | F05 | 3.2 |
| Unspecified fall | W19 | 3.2 |
| Superficial injury of head | S00 | 3.2 |
| Unspecified hematuria | R31 | 3.0 |
| Other bacterial agents as the cause of diseases classified to other chapters (secondary code) | B96 | 2.9 |
| Other symptoms and signs involving cognitive functions and awareness | R41 | 2.7 |
| Abnormalities of gait and mobility | R26 | 2.6 |
| Other cerebrovascular diseases | I67 | 2.6 |
| Convulsions, not elsewhere classified | R56 | 2.6 |
| Somnolence, stupor and coma | R40 | 2.5 |
| Complications of genitourinary prosthetic devices, implants, and grafts | T83 | 2.4 |
| Intracranial injury | S06 | 2.4 |
| Fracture of shoulder and upper arm | S42 | 2.3 |
| Other disorders of fluid, electrolyte, and acid-base balance | E87 | 2.3 |
| Other joint disorders, not elsewhere classified | M25 | 2.3 |
| Volume depletion | E86 | 2.3 |
| Senility | R54 | 2.2 |
| Care involving use of rehabilitation procedures | Z50 | 2.1 |
| Unspecified dementia | F03 | 2.1 |
| Other fall on same level | W18 | 2.1 |
| Problems related to medical facilities and other health care | Z75 | 2.0 |
| Vascular dementia | F01 | 2.0 |
| Superficial injury of lower leg | S80 | 2.0 |
| Cellulitis | L03 | 2.0 |
| Blindness and low vision | H54 | 1.9 |
| Deficiency of other B group vitamins | E53 | 1.9 |
| Problems related to social environment | Z60 | 1.8 |
| Parkinson's disease | G20 | 1.8 |
| Syncope and collapse | R55 | 1.8 |
| Fracture of rib(s), sternum and thoracic spine | S22 | 1.8 |
| Other functional intestinal disorders | K59 | 1.8 |
| Acute renal failure | N17 | 1.8 |
| Decubitus ulcer | L89 | 1.7 |
| Carrier of infectious disease | Z22 | 1.7 |
| Streptococcus and staphylococcus as the cause of diseases classified to other chapters | B95 | 1.7 |
| Ulcer of lower limb, not elsewhere classified | L97 | 1.6 |
| Other symptoms and signs involving general sensations and perceptions | R44 | 1.6 |
| Duodenal ulcer | K26 | 1.6 |
| Hypotension | I95 | 1.6 |
| Unspecified renal failure | N19 | 1.6 |
| Other septicemia | A41 | 1.6 |
| Personal history of other diseases and conditions | Z87 | 1.5 |
| Respiratory failure, not elsewhere classified | J96 | 1.5 |
| Exposure to unspecified factor | X59 | 1.5 |
| Other arthrosis | M19 | 1.5 |
| Epilepsy | G40 | 1.5 |
| Osteoporosis without pathological fracture | M81 | 1.4 |
| Fracture of femur | S72 | 1.4 |
| Fracture of lumbar spine and pelvis | S32 | 1.4 |
| Other disorders of pancreatic internal secretion | E16 | 1.4 |
| Abnormal results of function studies | R94 | 1.4 |
| Chronic renal failure | N18 | 1.4 |
| Retention of urine | R33 | 1.3 |
| Unknown and unspecified causes of morbidity | R69 | 1.3 |
| Other disorders of kidney and ureter, not elsewhere classified | N28 | 1.3 |
| Unspecified urinary incontinence | R32 | 1.2 |
| Other degenerative diseases of nervous system, not elsewhere classified | G31 | 1.2 |
| Nosocomial condition | Y95 | 1.2 |
| Other and unspecified injuries of head | S09 | 1.2 |
| Symptoms and signs involving emotional state | R45 | 1.2 |
| Transient cerebral ischemic attacks and related syndromes | G45 | 1.2 |
| Problems related to care-provider dependency | Z74 | 1.1 |
| Other soft tissue disorders, not elsewhere classified | M79 | 1.1 |
| Fall involving bed | W06 | 1.1 |
| Open wound of head | S01 | 1.1 |
| Other bacterial intestinal infections | A04 | 1.1 |
| Diarrhea and gastroenteritis of presumed infectious origin | A09 | 1.1 |
| Pneumonia, organism unspecified | J18 | 1.1 |
| Pneumonitis due to solids and liquids | J69 | 1.0 |
| Speech disturbances, not elsewhere classified | R47 | 1.0 |
| Vitamin D deficiency | E55 | 1.0 |
| Artificial opening status | Z93 | 1.0 |
| Gangrene, not elsewhere classified | R02 | 1.0 |
| Symptoms and signs concerning food and fluid intake | R63 | 0.9 |
| Other hearing loss | H91 | 0.9 |
| Fall on and from stairs and steps | W10 | 0.9 |
| Fall on same level from slipping, tripping, and stumbling | W01 | 0.9 |
| Thyrotoxicosis [hyperthyroidism] | E05 | 0.9 |
| Scoliosis | M41 | 0.9 |
| Dysphagia | R13 | 0.8 |
| Dependence on enabling machines and devices | Z99 | 0.8 |
| Agent resistant to penicillin and related antibiotics | U80 | 0.8 |
| Osteoporosis with pathological fracture | M80 | 0.8 |
| Other diseases of digestive system | K92 | 0.8 |
| Cerebral Infarction | I63 | 0.8 |
| Calculus of kidney and ureter | N20 | 0.7 |
| Mental and behavioral disorders due to use of alcohol | F10 | 0.7 |
| Other medical procedures as the cause of abnormal reaction of the patient | Y84 | 0.7 |
| Abnormalities of heartbeat | R00 | 0.7 |
| Unspecified acute lower respiratory infection | J22 | 0.7 |
| Problems related to life-management difficulty | Z73 | 0.6 |
| Other abnormal findings of blood chemistry | R79 | 0.6 |
| Personal history of risk-factors, not elsewhere classified | Z91 | 0.5 |
| Open wound of forearm | S51 | 0.5 |
| Depressive episode | F32 | 0.5 |
| Spinal stenosis | M48 | 0.5 |
| Disorders of mineral metabolism | E83 | 0.4 |
| Polyarthrosis | M15 | 0.4 |
| Other anemias | D64 | 0.4 |
| Other local infections of skin and subcutaneous tissue | L08 | 0.4 |
| Nausea and vomiting | R11 | 0.3 |
| Other noninfective gastroenteritis and colitis | K52 | 0.3 |
| Fever of unknown origin | R50 | 0.1 |

| **Table S3a:** *Streptococcus* species - distribution on species level | | |
| --- | --- | --- |
| **Microorganism** | **Counts** | **Percent** |
| **Non-hemolytic streptococci** |  |  |
| ***S. anginosus / S. milleri group*** |  |  |
| *S. anginosus* | 17 | 3.6 |
| *S. milleri* | 5 | 1.1 |
| *S. constellatus* | <3* | <0.6 |
| *S. intermedius* | <3 | <0.6 |
| ***S. bovis / equinus group*** |  |  |
| *S. bovis* | 33 | 7.0 |
| *S. gallolyticus* | 11 | 2.3 |
| *S. infantarius* | <3 | <0.6 |
| ***S. mitis group*** |  |  |
| *S. mitis* | 124 | 26.4 |
| *S. gordonii* | 19 | 4.0 |
| *S. oralis* | 9 | 1.9 |
| *S. parasanguinis* | <3 | <0.6 |
| *S. sanguinis* | 33 | 7.0 |
| *S. pneumoniae* | 26 | 5.5 |
| ***S. salivarius*** | 8 | 1.7 |
| ***S. mutans*** | 31 | 6.6 |
| **Non-hemolytic streptocci unspecified** | 37 | 7.9 |
| **Hemolytic streptococci** |  |  |
| Hemolytic streptococci unspecified | 3 | 0.6 |
| Hemolytic streptococci group A | 5 | 1.1 |
| Hemolytic streptococci group B | 40 | 8.5 |
| Hemolytic streptococci group C | 15 | 3.2 |
| Hemolytic streptococci group G | 24 | 5.1 |
| *Streptococcus dysgalactiae* | 3 | 0.6 |
| **Other** |  |  |
| *Abiotrophia defectiva* | 7 | 1.5 |
| *Gemella haemolysans* | <3 | <0.6 |
| *Granulicatella adiacens* | 3 | 0.6 |
| *Granulicatella elegans* | <3 | <0.6 |
| Streptococcus species unspecified | 8 | 1.7 |
| *Observations with a value below three is not allowed due to the data confidentiality policy of Statistics Denmark | | |

| **Table S3b:** Coagulase-negative staphylococci - distribution on species level | | |
| --- | --- | --- |
| **Microorganism** | **Counts** | **Percent** |
| *Staphylococcus capitis* | ≤3* | ≤3.1 |
| *Staphylococcus epidermidis* | 57 | 58.2 |
| *Staphylococcus haemolyticus* | ≤3 | ≤3.1 |
| *Staphylococcus hominis* | 5 | 5.1 |
| *Staphylococcus lugdunensis* | 15 | 15.3 |
| *Staphylococcus species unspecified* | 13 | 13.3 |
| *Staphylococcus warneri* | ≤3 | ≤3.1 |
| *Observations with a value below three is not allowed due to the data confidentiality policy of Statistics Denmark and you may not be able to calculate the exact value of a cell with a value below three. | | |

| **Table S3c:** Other microbiological causes - distribution on species level | | |
| --- | --- | --- |
| **Microorganism** | **Count** | **Percent** |
| **HACEK** |  |  |
| *Haemophilus parainfluenzae* | 5 | 7.0 |
| *Cardiobacterium hominis* | 7 | 9.9 |
| **Gram-positive bacteria** |  |  |
| *Aerococcus urinae* | 6 | 8.4 |
| **Gram-negative bacteria** |  |  |
| *Escherichia coli* | 8 | 11.3 |
| **Anaerobic** |  |  |
| *Cutibacterium acnes* | 8 | 11.3 |
| **Unspecified** | 3 | 4.2 |
| **Bacteria or fungi anonymized due to count below 3** | 34 | 47.9 |

| **Table S4:** Frequencies and proportions of the heart valves replaced in the surgical treated patients | | |
| --- | --- | --- |
| **Surgical valve replacements** | **Numbers** | **Proportion (%)** |
| **Left-sided valves** | **1189** | **92.8** |
| Isolated aortic valve* | 636 | 53.5 |
| Isolated mitral valve* | 344 | 28.9 |
| Combined (aortic and mitral valves) * | 209 | 17.6 |
| **Right-sided valves** | **31** | **2.4** |
| **Combined left -and right-sided valves** | **62** | **4.8** |
| Aortic valve and right-sided valves** | 28 | 45.2 |
| Mitral valve and right-sided valves** | 14 | 22.6 |
| Both aortic and mitral valves and right-sided valves** | 20 | 32.3 |
| *Frequencies and proportions of left-sided valve replacements. The percentages add up to 100% of all 1189 solely left-sided valve replacements.  **Frequencies and proportions of combined left -and right-sided valve replacements. The percentages add up to 100% of all 62 combined left -and right-sided valve replacements. | | |

| **Table S5:** Baseline characteristics of patients who survived until discharge | | | |
| --- | --- | --- | --- |
| Variable | Non-frail, N = 870 | Frail, N = 257 | p-value |
| Cumulative frailty score | 0.9 [0.0,2.4] | 7.7 [6.4,11.2] | <0.001 |
| Males | 685 (78.7%) | 186 (72.4%) | 0.032 |
| Age (years) | 63.0 [52.0,71.0] | 67.0 [55.0,73.0] | 0.003 |
| Length of hospital stay (days) | 46.0 [37.0,57.8] | 49.0 [43.0,64.0] | <0.001 |
| Microbiological etiology* |  |  | <0.001 |
| *S. aureus* | 159 (18.4%) | 66 (25.8%) |  |
| *Streptococcus* spp. | 343 (39.7%) | 75 (29.3%) |  |
| *Enterococcus* spp. | 116 (13.4%) | 55 (21.5%) |  |
| CoNS | 69 (8.0%) | 21 (8.2%) |  |
| Other | 53 (6.1%) | 11 (4.3%) |  |
| Negative | 125 (14.5%) | 28 (10.9%) |  |
| Prior prosthesis | 136 (15.6%) | 69 (26.8%) | <0.001 |
| Cardiac implantable electrical devices (CIED) | 40 (4.6%) | 19 (7.4%) | 0.077 |
| Aortic valve disease | 218 (25.1%) | 105 (40.9%) | <0.001 |
| Mitral valve disease | 78 (9.0%) | 37 (14.4%) | 0.011 |
| Atrial fibrillation (AF) | 98 (11.3%) | 54 (21.0%) | <0.001 |
| Heart failure (HF) | 86 (9.9%) | 49 (19.1%) | <0.001 |
| Ischemic heart disease (IHD) | 107 (12.3%) | 69 (26.8%) | <0.001 |
| Hypertension | 285 (32.8%) | 137 (53.3%) | <0.001 |
| Chronic kidney disease (CKD) | 22 (2.5%) | 41 (16.0%) | <0.001 |
| Dialysis | 16 (1.8%) | 25 (9.7%) | <0.001 |
| Diabetes | 88 (10.1%) | 67 (26.1%) | <0.001 |
| Liver disease | 11 (1.3%) | 18 (7.0%) | <0.001 |
| Chronic obstructive pulmonary disease (COPD) | 39 (4.5%) | 23 (8.9%) | 0.006 |
| Malignancy | 67 (7.7%) | 32 (12.5%) | 0.018 |
| Charlson cormorbidity index |  |  | <0.001 |
| 0 | 583 (67.0%) | 70 (27.2%) |  |
| 1-2 | 239 (27.5%) | 116 (45.1%) |  |
| >2 | 48 (5.5%) | 71 (27.6%) |  |
| Anticoagulants | 126 (14.5%) | 81 (31.5%) | <0.001 |
| Beta blockers | 179 (20.6%) | 93 (36.2%) | <0.001 |
| Lipid-lowering medication | 211 (24.3%) | 118 (45.9%) | <0.001 |
| RAS inhibitors | 263 (30.2%) | 121 (47.1%) | <0.001 |
| *6 Missing values  Categorical variables were reported with frequency and percentages and continues variables with median and 25th-75th percentiles. To assess the statistical difference between the two groups, we used the Pearson’s Chi-square test for categorical variables and the non-parametric Wilcoxon rank sum test for continues variables. | | | |

| **Table S6:** Summary of events, cumulative follow-up time and crude event rates for all outcomes stratified by frailty. | | | |
| --- | --- | --- | --- |
| **Outcomes** | Number of events / number of patients | Total follow-up in person years | Event rate per 1000 person years with corresponding 95% confidence intervals* |
| *Frail patients* |  |  |  |
| Death or rehospitalization (≥14 days) | 137 / 315 | 217.5 | 629.8 [532.7-744.6] |
| All-cause mortality | 85/315 | 251.2 | 338.3 [273.5-418.5] |
| Rehospitalization (≥14 days)** | 68 /257 | 201.1 | 338.2 [266.7-429.0] |
| *Patients with low frailty scores* |  |  |  |
| Death or rehospitalization (≥14 days) | 252 / 967 | 784.5 | 321.2 [283.9-363.4] |
| All-cause mortality | 145 / 967 | 846.9 | 171.2 [145.5-201.5] |
| Rehospitalization (≥14 days)** | 142/870 | 755.6 | 187.9 [159.4-221.5] |
| * Incidence rates and corresponding 95% confidence intervals was calculated with Poisson regression  **For the outcome rehospitalization follow-up began from IE discharge, thus patients who died during admission were excluded (N=155) | | | |

| **Table S7:** Baseline characteristics according to frailty in three groups | | | | |
| --- | --- | --- | --- | --- |
| Variable | Low frailty, N = 967 | Intermediate frailty, N = 270 | High frailty, N = 45 | p-value |
| Cumulative frailty score | 1.1 [0.0,2.5] | 7.4 [6.2,9.7] | 17.6 [16.2,21.9] | <0.001 |
| Males | 753 (77.9%) | 188 (69.6%) | 38 (84.4%) | 0.008 |
| Age (years) | 64.0 [53.0,72.0] | 68.0 [58.0,73.0] | 57.0 [49.0,73.0] | <0.001 |
| Length of hospital stay (days) | 45.0 [34.0,57.0] | 48.0 [40.0,63.8] | 53.0 [44.0,65.0] | <0.001 |
| Microbiological etiology* |  |  |  | <0.001 |
| *S. aureus* | 187 (19.4%) | 70 (25.9%) | 15 (34.1%) |  |
| *Streptococcus* spp. | 381 (39.6%) | 82 (30.4%) | 7 (15.9%) |  |
| *Enterococcus* spp. | 125 (13.0%) | 56 (20.7%) | 13 (29.5%) |  |
| CoNS | 72 (7.5%) | 20 (7.4%) | 6 (13.6%) |  |
| Other | 57 (5.9%) | 13 (4.8%) | <3 |  |
| Negative | 140 (14.6%) | 29 (10.7%) | <3 |  |
| Prior prosthesis | 158 (16.3%) | 78 (28.9%) | 13 (28.9%) | <0.001 |
| Cardiac implantable electrical devices (CIED) | 46 (4.8%) | 22 (8.1%) | 3 (6.7%) | 0.082 |
| Aortic valve disease | 244 (25.2%) | 102 (37.8%) | 20 (44.4%) | <0.001 |
| Mitral valve disease | 83 (8.6%) | 39 (14.4%) | 4 (8.9%) | 0.019 |
| Atrial fibrillation (AF) | 120 (12.4%) | 63 (23.3%) | 8 (17.8%) | <0.001 |
| Heart failure (HF) | 97 (10.0%) | 54 (20.0%) | 9 (20.0%) | <0.001 |
| Ischemic heart disease (IHD) | 134 (13.9%) | 78 (28.9%) | 15 (33.3%) | <0.001 |
| Hypertension | 343 (35.5%) | 150 (55.6%) | 22 (48.9%) | <0.001 |
| Chronic kidney disease (CKD) | 24 (2.5%) | 41 (15.2%) | 13 (28.9%) | <0.001 |
| Dialysis | 18 (1.9%) | 24 (8.9%) | 11 (24.4%) | <0.001 |
| Diabetes | 116 (12.0%) | 74 (27.4%) | 12 (26.7%) | <0.001 |
| Liver disease | 14 (1.4%) | 18 (6.7%) | 3 (6.7%) | <0.001 |
| Chronic obstructive pulmonary disease (COPD) | 44 (4.6%) | 27 (10.0%) | 6 (13.3%) | <0.001 |
| Malignancy | 72 (7.4%) | 33 (12.2%) | 7 (15.6%) | 0.012 |
| Charlson cormorbidity index |  |  |  | <0.001 |
| 0 | 634 (65.6%) | 71 (26.3%) | 5 (11.1%) |  |
| 1-2 | 276 (28.5%) | 125 (46.3%) | 15 (33.3%) |  |
| >2 | 57 (5.9%) | 74 (27.4%) | 25 (55.6%) |  |
| Anticoagulants | 157 (16.2%) | 90 (33.3%) | 14 (31.1%) | <0.001 |
| Beta blockers | 217 (22.4%) | 103 (38.1%) | 19 (42.2%) | <0.001 |
| Lipid-lowering medication | 262 (27.1%) | 128 (47.4%) | 23 (51.1%) | <0.001 |
| RAS inhibitors | 313 (32.4%) | 135 (50.0%) | 19 (42.2%) | <0.001 |

*6 Missing values

Categorical variables were reported with frequency and percentages and continues variables with median and 25th-75th percentiles. To assess the statistical difference between the two groups, we used the Pearson’s Chi-square test for categorical variables and the non-parametric Wilcoxon rank sum test for continues variables.

| **Table S8:** Baseline Characteristics according to frailty in elderly patients (≥75 years of age) | | | |
| --- | --- | --- | --- |
| Variable | Patients with low frailty scores,  N = 155 | Frail patients,  N = 62 | p-value |
| Cumulative frailty score | 1.4 [0-2.9] | 7.9 [6.7 – 10.1] | <0.001 |
| Males | 109 (70.3%) | 42 (67.7%) | 0.709 |
| Age (years) | 78 [76-79] | 77 [75-80] | 0.805 |
| Length of hospital stay (days) | 48 [36 -62] | 48 [39-56] | 0.856 |
| Microbiological etiology |  |  | 0.444 |
| *S. aureus* | 19 (12.3%) | 12 (19.4%) |  |
| *Streptococcus* spp. | 53 (34.2%) | 17 (27.4%) |  |
| *Enterococcus* spp. | 40 (25.8%) | 21 (33.9%) |  |
| CoNS | 13 (8.4%) | 5 (8.1%) |  |
| Other | 11 (7.1%) | <3 |  |
| Negative | 19 (12.3%) | 5 (8.1%) |  |
| Prior prosthesis | 44 (28.4%) | 24 (38.7%) | 0.139 |
| Cardiac implantable electrical devices (CIED) | 15 (9.7%) | 9 (14.5%) | 0.305 |
| Aortic valve disease | 61 (39.4%) | 34 (54.8%) | 0.038 |
| Mitral valve disease | 11 (7.1%) | 9 (14.5%) | 0.088 |
| Atrial fibrillation (AF) | 36 (23.2%) | 18 (29.0%) | 0.371 |
| Heart failure (HF) | 26 (16.8%) | 14 (22.6%) | 0.319 |
| Ischemic heart disease (IHD) | 41 (26.5%) | 26 (41.9%) | 0.026 |
| Hypertension | 77 (49.7%) | 33 (53.2%) | 0.637 |
| Chronic kidney disease (CKD) | 4 (2.6%) | 9 (14.5%) | 0.002 |
| Dialysis | <3 | 6 (9.7%) | 0.008 |
| Diabetes | 18 (11.6%) | 20 (32.3%) | <0.001 |
| Liver disease | <3 | 3 (4.8%) | 0.071 |
| Chronic obstructive pulmonary disease (COPD) | 15 (9.7%) | 7 (11.3 %) | 0.722 |
| Malignancy | 20 (12.9%) | 11 (17.7%) | 0.357 |
| Charlson comorbidity index |  |  | <0.001 |
| 0 | 81 (52.3%) | 18 (29.0%) |  |
| 1-2 | 64 (41.3%) | 27 (43.5%) |  |
| >2 | 10 (6.5%) | 17 (27.4%) |  |
| Anticoagulants | 42 (27.1%) | 21 (33.9%) | 0.321 |
| Beta blockers | 43 (27.7%) | 23 (37.1%) | 0.176 |
| Lipid-lowering medication | 62 (40.0%) | 41 (66.1%) | <0.001 |
| RAS inhibitors | 65 (41.9%) | 28 (45.2%) | 0.664 |
| Categorical variables were reported with frequency and percentages and continues variables with median and 25th-75th percentiles. To assess the statistical difference between the two groups, we used the Pearson’s Chi-square test for categorical variables and the non-parametric Wilcoxon rank sum test for continues variables | | | |

**Fig. S1: Days hospitalized within one year from discharge according to frailty**

**
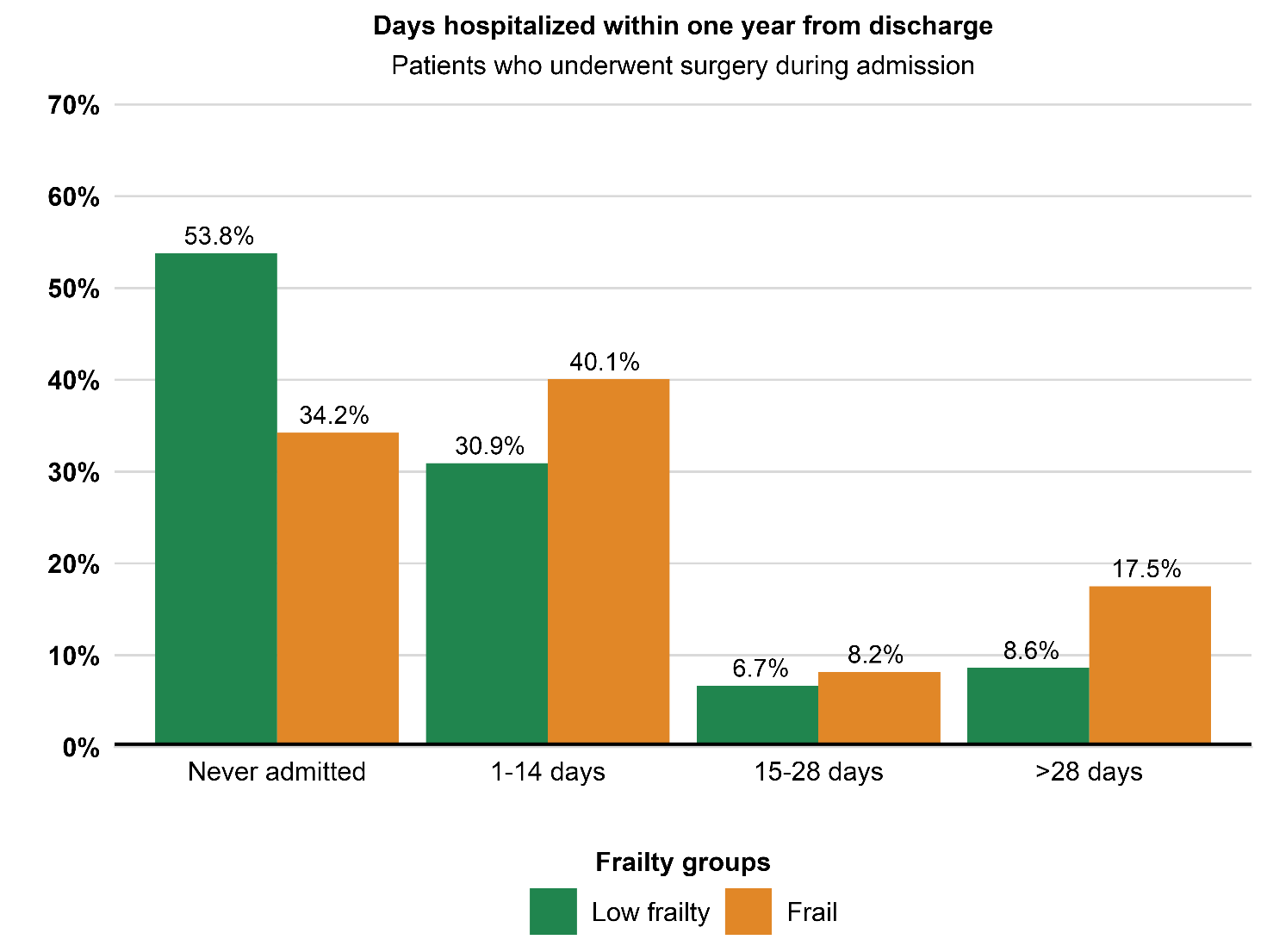
**

**Fig. S2: Days hospitalized or mortality within one year from discharge according to frailty (low, intermediate, and high frailty)**

**
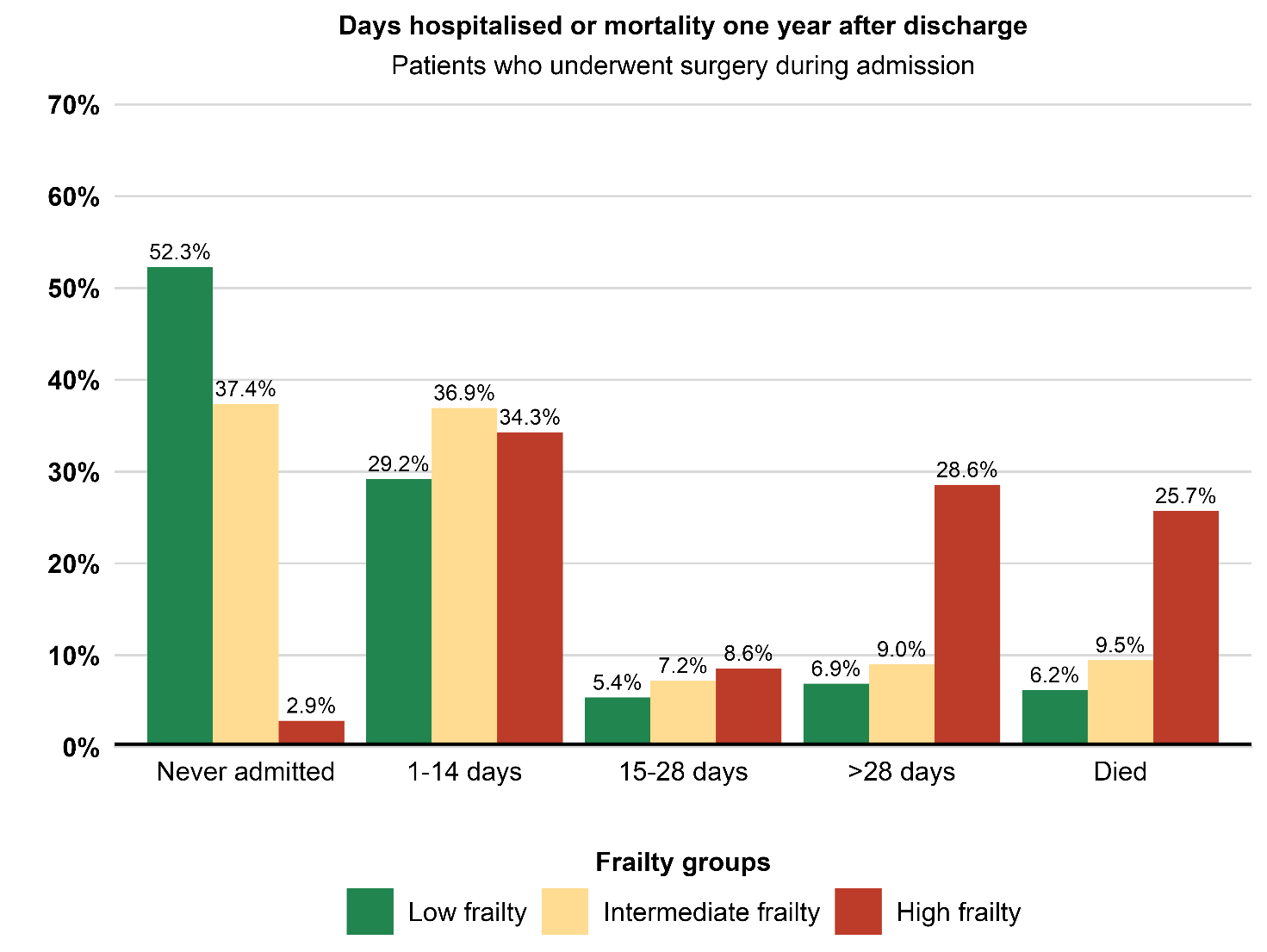
**

**Fig. S3a-c: Crude risk of rehospitalization or death during 12 months of follow-up according to frailty (low, intermediate, and high frailty)**


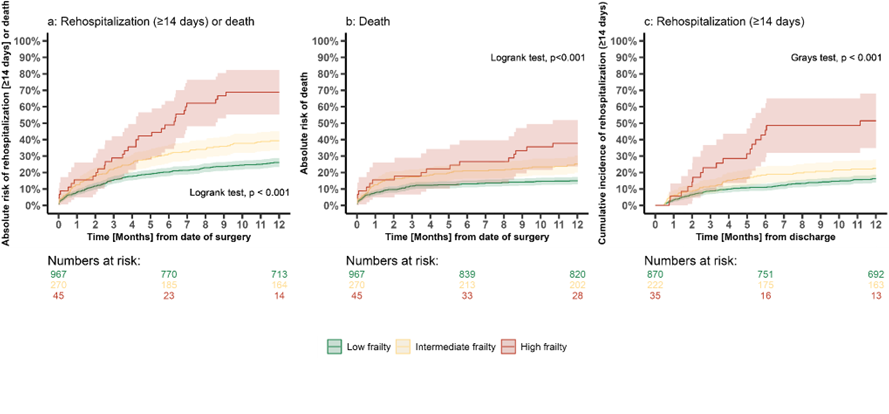

Supplement: Supplementary file 1 — Supplementary file1 (DOCX 530 KB) [file 15010_2024_2262_MOESM1_ESM.docx]
